# Where Shall We Spend the Summer?

**Published:** 1875-07

**Authors:** 


### WHERE SHALL WE SPEND THE SUMMER?

Thousands have been asking themselves this question lately, and we are of that number. In reflecting upon the subject, it struck us that we might, by a little pleasant effort, gain the needed information, and prepare ourself to give a little advice to our friends. So we started out as soon as the June number was fairly launched, on a tour of observation.

The mountains of New Hampshire were our objective point, because we dwellers by the sea do better if we go away from saline influences during a part of the year. We determined that we would explore a section new to us—the country between Portland, Maine, and the notch of the White Mountains. The easiest way to do this was to proceed to Boston. So we took that delightful trip in the grand steamer Bristol, of the Old Colony line, and sailed out one moonlit night, on the great Sound, which was as calm as a Summer lake. The Eastern Railroad conveyed us from Boston to Portland, where we dined at as fine a hotel as any city can boast of—the "Falmouth." At 2 P.M. we took train on the "Portland & Ogdensburg" road for North Conway, 60 miles away. We made the run in two and a half hours, through a beautiful country, green and wooded and rolling, the landscape interspersed with lakes and rivers and roaring cascades. Sebago Lake, a beautiful sheet of water, on which two steamers ply, is about an hour's ride from Portland, and is a very attractive spot. In fact, the road is lined with picturesque, natural objects. We pass the Glen Station, where stages receive passengers for the popular "Glen House," one of the best hotels, it is said, in the mountains. It is kept by the Messrs. Milliken, wholesale merchants in Portland, who have the ability, financial and otherwise, to "keep a hotel" as it should be kept. Their house is reached, also, by stage from Gorham, on the Grand Trunk road.

North Conway was painting and polishing up for the Summer campaign. The great "Kiarsarge House" looked somewhat deserted, though a few guests had arrived as early as the 20th of May. The Misses Thompson, the proprietors, were anticipating a good season, and the other hotel-keepers seemed to regard the outlook as promising. At the "Intervale House," North Conway, N. H., we found Mr. Stephen Mudgett and his sons, the proprietors, engaged in putting the place in perfect order, and all looked as bright as a new pin. The view from this house is as perfect as could be wished, embracing a vast expanse of valley and mountain scenery. Mr. Mudgett informed us that his terms were from $1.50 to $2.50 per day, according to location of rooms; and the reputation of the house is excellent all over the region. We *underscored* our memoranda here, and decided that this would be a good place to see again. The Eastern railroad has a branch to this place.

Then we went on by the Portland & Ogdensburg railroad to Upper Bartlett, 12 miles, which takes us pretty well up towards the Notch. Here we saw the Bartlett House, the only hotel, where Mr. Frank George entertains, in a comfortable way, about fifty guests during the Summer, at rates ranging from $7 to $12 per week; a good place, we decided, to combine economy with grand mountain scenery and good, wholesome, substantial living.

We kept on to Bemis, six miles above Bartlett, beyond which the railroad did not then take passengers, though the iron was laid 3 or 4 miles beyond. We took coach for the Crawford House, nine miles above. Passing through the Notch we saw the railroad grade high up on the mountain-side. It has been a great engineering work to construct this portion of the road, and multitudes will be attracted to the region to gaze down the rugged abysses along which it winds its secure though devious way. It is to be running through to connect with the Boston, Concord & Montreal road at Fabyan's during the Summer.

We tarried for the night at the Crawford, where we also found painters and whitewashers and decorators at work, in charge of Mr. Merrill, the manager of the house, under the Barrons, who own this, as well as the "Twin Mountain House," and the "Junction House," at White River, Vermont. The Crawford was being put in superb condition, and the evidences were that it would be even more popular the present season than ever.

We looked in at the great "Fabyan House," kept by Lindsay & French, and here, too, the note of preparation was being sounded in all directions. The house is, we were told, the largest in the mountains, and it certainly is a very perfect establishment in all its appointments. Here we took the cars of the Boston, Concord & Montreal railroad, and pushed on to the "Twin Mountain House"—the favorite Summer home of Mr. Beecher, whose great popularity, of course, attracts a host of visitors. The Messrs. Barron are remarkable men as well as remarkable hotel-keepers. They run great saw-mills, and make choice lumber for Chickering's pianos, and many carriage-builders. They freeze hundreds of tons of fat turkeys in winter, and keep them frozen till summer, when they supply them fresh and plump and sweet to the guests of their hotels; or, if they can spare them, they send car-loads to the great markets of New York and Boston, where Barron's frozen turkies have been a choice and staple commodity for 18 years. They own about 1200 acres of choice land near White River, and cultivate great gardens such as can be seen nowhere else. Thus, nearly all their farm and garden supplies are raised under their own eyes, and the patrons of their three fine hotels reap the advantages of their foresight and intelligence.

At Bellows' Falls, Vermont—a lovely spot, situated at the very base of the majestic Mount Kilbonne, we found the Messrs. Towns' father and son, preparing their fine hotel—The Island House—for the summer guests, who are always numerous here. The natural attractions are many, and intelligent art has ably supplemented them. The hotel is of brick, and is situated on an island in the lovely Connecticut River. A bridge connects it with the main land. Fountains play around the house, and a delightful breeze is felt at all times. The table is excellent, as all testify, and the rooms are large. The price is from $10 to $17 per week. About 180 guests can be comfortably housed. Mrs. Towns, a lady of excellent taste and great energy of character, personally superintends many of the details of the household. We were very favorably impressed with this house, as well as with Town Hotel owned by the same family and kept open all the year round. If we do not find ourselves at the "Island" again this Summer, our calculations will be at fault.

At Littleton, on the Boston, Concord & Montreal raiload, there are two hotels, "Thayer's Hotel" and the "Oak Hill House"—the latter open only during the summer, and very pleasantly situated. The house kept by Mr. H. L. Thayer, has been a popular resort with the traveling public for twenty-five years, and its reputation is first-class. It accommodates 150 guests, and is always well filled in summer. It keeps open the year round. The charges are moderate. Littleton is a very attractive spot, the lovely Amanoosuc River dividing the town, and the country being rugged and mountainous. Many tourists, bound for the Franconia and White Mountains, prefer to tarry awhile here and make excursions to the various points of interest. From here to the Profile, the distance is 12 miles, over a lovely mountain road. Here reside the Brothers Kilburn, whose photograhic views of natural scenery are so famous, and who have here the largest photographic establishment in America.

Other Summer resorts were visited in our little trip, but we are compelied to defer notice of them until our next number, which will be ready for delivery on the 10th of July. By that time we hope to be able to answer the question—Where shall we spend our Summer? Reasonable prices appear to prevail all through the mountains, and this is a great consolation in hard times.

---

The summer hat should be light in texture, light in color and have a high crown. Space for air between the head and the crown of the hat is most important, since a low crown in contact with the head affords but slight protection from the rays of the sun. The high straw hats with ventilating openings between the braids are cool and comfortable.

The light, white, high-crown hat, "stove pipe," is also comfortable, protects well from the sun, and is considered more "dressy" than the straw.

Many laboring men wear low felt hats, or cloth caps in the hotest weather. It is very natural that they should be the most common victims of sun-stroke.

---

The generality of men spend the early part of their lives in contributing to render the latter part miserable.

